# Primary HIV Infection Presenting As Inflammatory Polyradiculopathy Mimicking Guillain-Barré Syndrome: A Case Report

**DOI:** 10.7759/cureus.104087

**Published:** 2026-02-22

**Authors:** Mohammed Khalid, SureshKumar Rasiah

**Affiliations:** 1 General Medicine, Armadale Health Services, Armadale, AUS; 2 Internal Medicine, Amina Hospital, Ajman, ARE; 3 General Medicine and Nephrology, Armadale Health Services, Armadale, AUS

**Keywords:** cauda equina, guillain-barré syndrome, hiv, neuropathy, polyradiculopathy

## Abstract

Primary HIV infection rarely presents with acute neuromuscular weakness. In patients with polyradiculopathy, HIV infection should be considered even in the absence of recognized risk factors. We report a 36-year-old woman with rapidly progressive paraparesis who was initially suspected to have Guillain-Barré syndrome (GBS). Subsequent investigations confirmed an HIV-associated inflammatory polyradiculopathy. This case highlights diagnostic challenges and emphasizes the need for routine HIV testing in unexplained radiculopathies.

## Introduction

Acute lower limb weakness encompasses a wide diagnostic spectrum, including inflammatory neuropathies, infectious radiculopathies, metabolic disorders, and structural spinal disease. One important cause is Guillain-Barré syndrome (GBS), an acute post-infectious autoimmune neuropathy characterized by rapidly progressive symmetric weakness, reduced or absent reflexes (areflexia), and elevated cerebrospinal fluid (CSF) protein with few or no inflammatory cells (albuminocytologic dissociation). GBS typically follows a viral or bacterial infection and is one of the most common causes of acute flaccid paralysis worldwide [1].

Polyradiculopathy refers to inflammation affecting multiple spinal nerve roots, leading to weakness, pain, and reduced reflexes. In advanced HIV infection, lumbosacral polyradiculopathy most commonly results from opportunistic infections such as cytomegalovirus (CMV), followed by neurosyphilis and lymphomatous meningitis [2]. However, neurological complications may also occur during early or primary HIV infection, when the virus first enters the nervous system.

Primary HIV-associated inflammatory polyradiculopathy is rare, occurring in less than 1% of acute HIV presentations, but it can closely mimic GBS due to overlapping features such as symmetric weakness and areflexia [3]. Unlike GBS, which typically shows elevated CSF protein with minimal cells, HIV-associated polyradiculopathy often demonstrates lymphocytic pleocytosis, reflecting direct viral-associated inflammation of the nerve roots. With the widespread use of antiretroviral therapy, such presentations have become less frequent; however, they remain an important diagnostic consideration, particularly in patients without recognized HIV risk factors [4].

Here, we describe a case of primary HIV infection presenting as inflammatory polyradiculopathy, initially suspected to be GBS, highlighting the diagnostic challenges and the importance of early HIV testing in unexplained acute radiculopathy.

## Case presentation

A 36-year-old woman presented with sudden-onset bilateral lower limb weakness for two days, more pronounced on the left. She described heaviness in both legs, gait unsteadiness, and bilateral leg pain radiating to the ankles for two weeks. She denied sensory loss. A self-limiting upper respiratory tract infection had occurred two weeks prior. She reported weight loss and reduced appetite over four months. Her past medical history included mild sickle cell disease. She had no recent travel history, denied intravenous drug use, and reported no recent sexual activity.

On examination, she had bilateral cervical lymphadenopathy and an antalgic gait requiring a walking frame. Lower limb strength was 3/5 bilaterally, with preserved upper limb power. Sensation to pain, temperature, and proprioception was normal. Deep tendon reflexes were absent in the lower limbs, and plantar responses were flexor.

Initial investigations revealed a mild systemic inflammatory response, with a C-reactive protein (CRP) of 15 mg/L and a slightly elevated alanine transaminase (ALT) of 53 U/L, while bilirubin was normal at 8 µmol/L (Table 1). Neuroimaging via MRI of the lumbosacral spine demonstrated diffuse contrast enhancement of the cauda equina nerve roots in the absence of spinal canal stenosis.

**Table 1:** Blood investigations with normal reference range CRP: C-reactive protein; ALT: alanine transaminase

Investigation	Patient result	Reference range (adult)
CRP	15 mg/L	<5 mg/L
ALT	53 U/L	7-55 U/L
Bilirubin	8 µmol/L	3-21 µmol/L

Lumbar puncture confirmed significant central nervous system involvement, showing marked lymphocytic pleocytosis of 229 cells/µL and an elevated CSF protein of 1.76 g/L, with a CSF glucose of 3.2 mmol/L. Initial screening for cryptococcal antigen and herpes simplex virus (HSV)/varicella zoster virus (VZV) polymerase chain reaction (PCR) was negative (Table 2). The broad differential diagnosis, spanning infectious, autoimmune, and neoplastic causes, was clarified when HIV serology returned positive. Further investigations, including CSF analysis, MRI findings, and the clinical response to antiretroviral therapy, supported the diagnosis, as discussed below. Advanced virological studies confirmed a high plasma HIV viral load of 890000 copies/mL and a CSF HIV viral load of 34000 copies/mL. Immunological profiling showed a cluster of differentiation 4 (CD4) count of 356 cells/mm³ and a severely inverted CD4/CD8 ratio of 0.17, suggesting an acute or advanced HIV-associated neurological syndrome (Tables 3, 4). 

**Table 2:** CSF analysis CSF: cerebrospinal fluid; TB: tuberculosis; CMV: cytomegalovirus; PCR: polymerase chain reaction; HSV: herpes simplex virus; VZV: varicella zoster virus

Investigation	Patient result	Reference range (adult)
CSF white blood cell count	229 cells/µL (lymphocyte predominant)+1	0-5 cells/µL+1
CSF protein	1.76 g/L+1	0.15-0.45 g/L+1
CSF glucose	3.2 mmol/L	2.2-3.9 mmol/L
CSF cryptococcal antigen	Negative	Negative
CSF CMV PCR	Negative	Negative
CSF treponema pallidum (syphilis)	Negative	Negative
CSF TB PCR	Negative	Negative
CSF HSV/VZV PCR	Negative	Negative

**Table 3:** Virology and immunology investigation CSF: cerebrospinal fluid; CD: cluster of differentiation

Investigation	Patient result	Reference range (adult)
HIV serology	Positive	Negative
Plasma HIV viral load	890000 copies/mL	Undetectable
CSF HIV viral load	34000 copies/mL	Undetectable
CD4 T-cell count	356 cells/mm³	500-1500 cells/mm³
CD4/CD8 ratio	0.17	1.0-4.0

**Table 4:** Other viral serologies CMV: cytomegalovirus

Test	Result
Hepatitis B surface antigen	Positive
Chlamydia serology	Negative
Gonorrhoea serology	Negative
CMV serology	Negative
Toxoplasma serology	Negative

**Figure 1:**
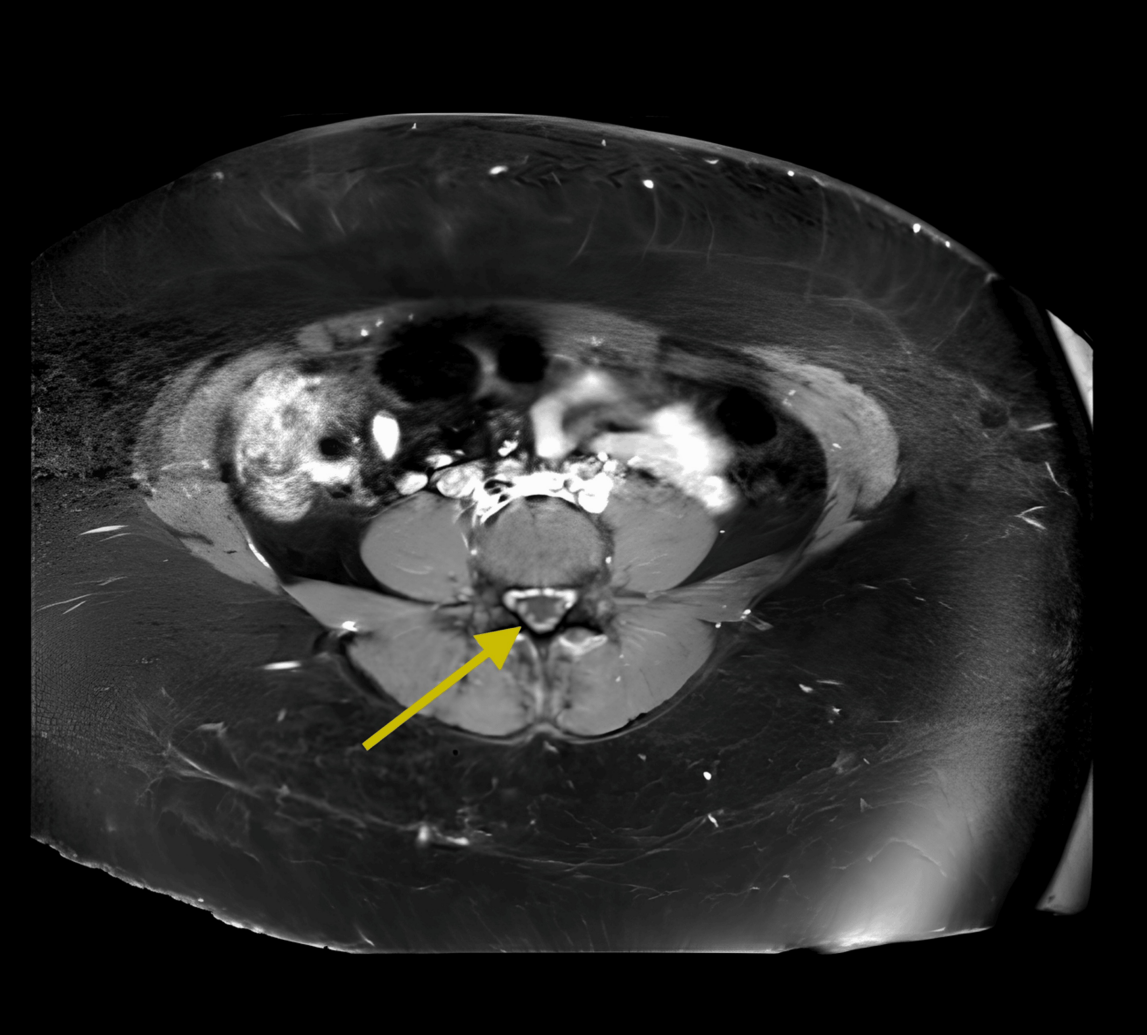
Contrast-enhanced MRI of the lumbosacral spine demonstrating diffuse enhancement of the cauda equina nerve roots, consistent with inflammatory polyradiculopathy T1-weighted image with gadolinium contrast showing enhancement of the cauda equina nerve roots.

Electromyography (EMG) showed no demyelinating features. A CT scan of the chest, abdomen, and pelvis demonstrated widespread lymphadenopathy. A repeat MRI of the spine showed persistent ventral cauda equina nerve root enhancement (Figure 1).

A diagnosis of HIV-associated inflammatory polyradiculopathy was made. The patient received three days of intravenous methylprednisolone. Antiretroviral therapy (Biktarvy) was commenced, providing dual activity against HIV and hepatitis B. Due to persistent weakness after three weeks, intravenous immunoglobulin (IVIG) was initiated, with modest improvement.

Following IVIG therapy (0.4 g/kg/day for five days), neurological examination at discharge (week 4) showed improvement in lower limb strength from 3/5 to 4/5 bilaterally on the Medical Research Council (MRC) scale. The patient was able to ambulate short distances with assistance. The plasma HIV viral load demonstrated a significant reduction following initiation of antiretroviral therapy, consistent with an early treatment response. She was discharged after four weeks with mild residual weakness and was arranged for home rehabilitation.

Long-term follow-up CD4 count and CSF parameters were not available at the time of manuscript preparation, as the patient transitioned to outpatient care. However, an early clinical and virological response following antiretroviral therapy (ART) initiation was observed. Table 5 presents the timeline of clinical events.

**Table 5:** Timeline of clinical events IVIG: intravenous immunoglobulin; CSF: cerebrospinal fluid; ART: antiretroviral therapy

Time	Event
4 months prior	Weight loss and reduced appetite
2 weeks prior	Self-limiting upper respiratory tract infection
2 weeks prior	Onset of bilateral radicular leg pain
2 days prior	Sudden bilateral lower limb weakness and gait instability
Day 0	Hospital presentation; examination showed bilateral lower limb weakness (3/5), areflexia, preserved sensation, and cervical lymphadenopathy
Days 1-3	Blood tests: CRP elevated; MRI spine showed diffuse cauda equina enhancement; CSF revealed lymphocytic pleocytosis and elevated protein
Day 4	HIV was diagnosed; plasma viral load 890000 copies/mL; CSF viral load 34000 copies/mL; CD4 356 cells/mm³
Week 1	IV methylprednisolone initiated
Weeks 1-2	ART (Biktarvy) commenced
Week 3	Persistent weakness noted
Weeks 3-4	IVIG was administered with modest improvement
Week 4	Discharged with mild residual weakness; rehabilitation was arranged

## Discussion

Primary HIV infection presenting as polyradiculopathy is uncommon. HIV-associated neuropathies include distal symmetric polyneuropathy, GBS during seroconversion, chronic inflammatory demyelinating polyneuropathy (CIDP), and radiculopathies [5]. Distinguishing HIV-associated radiculopathy from GBS is essential, given the differences in pathophysiology and management.

CMV polyradiculopathy typically occurs in advanced immunosuppression (CD4 <50 cells/mm³), with polymorphonuclear CSF pleocytosis, severe sphincter dysfunction, and rapid neurological decline [5]. In certain instances, CMV can be identified in the CSF. However, due to false-negative results and the possibility that viral cultures may remain negative for up to a week, viral culture should not be used as the sole diagnostic method [5]. In contrast, this patient had moderate immunosuppression (CD4 count 356 cells/mm³), lymphocytic CSF pleocytosis, and no bladder involvement, supporting an immune-mediated HIV radiculitis rather than CMV infection.

MRI is valuable for detecting nerve root inflammation [6]. Cauda equina enhancement, as demonstrated in this case, strongly supports inflammatory polyradiculopathy. EMG findings may be normal in early or dorsal root-predominant radiculitis [7]. Table 6 presents a comparison of GBS and HIV-associated inflammatory polyradiculopathy [8,9].

**Table 6:** Comparison of GBS and HIV-associated inflammatory polyradiculopathy GBS: Guillain-Barré syndrome; CSF: cerebrospinal fluid; EMG: electromyography; CD: cluster of differentiation

Feature	GBS	HIV-associated polyradiculopathy
CSF cell count	Normal or mild ↑ (albuminocytologic dissociation)	Marked lymphocytic pleocytosis
CSF protein	Elevated	Elevated
MRI findings	Often normal or mild root enhancement	Pronounced cauda equina enhancement
Typical onset	Rapid, post-infectious	Acute or subacute, variable
EMG findings	Demyelinating or axonal changes	May be normal early
Immune status	Normal	May occur even with moderate CD4 count

The distinction between HIV-associated radiculopathy and GBS is essential due to differing management strategies. In this case, the patient’s moderate CD4 count (356 cells/mm³) and lymphocytic pleocytosis were inconsistent with the neutrophilic pleocytosis and profound immunosuppression (CD4 <50 cells/mm³) typically seen in CMV-associated polyradiculopathy [9]. Furthermore, MRI findings of diffuse contrast enhancement of the cauda equina nerve roots provided objective evidence of an inflammatory process. While EMG may be normal in early or dorsal root-predominant stages, the integration of high plasma and CSF viral loads provides strong supportive evidence that HIV was the primary driver (Table 7).

**Table 7:** Systemic exclusion of competing diagnosis CMV: cytomegalovirus; CSF: cerebrospinal fluid; PCR: polymerase chain reaction; HSV: herpes simplex virus; VZV: varicella zoster virus; CD: cluster of differentiation; TB: tuberculosis

Competitor diagnosis	Evidence for exclusion
CMV radiculitis	Excluded due to CD4 count of 356 cells/mm³ (typically <50 cells/mm³ in CMV), lymphocyte-predominant pleocytosis (vs. polymorphonuclear pleocytosis), and negative CMV serology and PCR
Neurosyphilis and TB	Included in the initial broad differential; routine blood tests and CSF analysis showed no supportive features (e.g., negative routine screening). CSF PCR for *Treponema pallidum* and *Mycobacterium tuberculosis* was negative
Leptomeningeal malignancy	Ruled out by CT of the chest, abdomen, and pelvis, which showed widespread lymphadenopathy but no evidence of a primary solid tumor or focal malignancy
HSV/VZV/cryptococcus	Ruled out by negative CSF PCR and cryptococcal antigen testing

Long-term outcomes in HIV-associated polyradiculopathy are favorable with early ART, with more than 70% achieving full recovery within six to 12 months, although residual deficits occur in 20%- 30% of cases [10]. In this case, longer-term follow-up would be required to assess sustained improvement. Treatment for HIV-associated polyradiculopathy is not well established. Corticosteroids may reduce inflammation, and early initiation of ART is essential. IVIG may be considered in cases with overlapping immune-mediated neuropathic features or diagnostic uncertainty [11].

Antiretroviral therapy with bictegravir/emtricitabine/tenofovir alafenamide (Biktarvy) was initiated, providing effective treatment for both HIV and concomitant hepatitis B infection. Neurological improvement was observed following initiation of antiretroviral therapy, corticosteroids, and IVIG. However, given the single-case design, it is not possible to attribute recovery to antiretroviral therapy alone. The observed improvement likely reflects the combined effects of immunomodulatory treatment, supportive care, and the natural course of immune-mediated inflammatory radiculopathy.

Despite initial corticosteroid therapy, lower limb weakness persisted at 3/5 strength after three weeks, prompting IVIG administration. Following IVIG and continued ART, neurological function improved to 4/5 bilaterally by discharge, supporting an immune-mediated inflammatory process responsive to immunomodulatory therapy and viral suppression.

## Conclusions

This case demonstrates inflammatory polyradiculopathy occurring in the setting of previously undiagnosed HIV infection with CSF viral involvement. The temporal association between untreated HIV infection and inflammatory neurological findings, in the absence of alternative infectious or structural causes, supports a plausible relationship between HIV infection and the observed neurological syndrome. However, given the observational nature of a single case and the use of concurrent immunomodulatory and supportive therapies, a definitive causal relationship between HIV infection and neurological dysfunction, or attribution of neurological recovery to specific treatments, cannot be established.

This report highlights the importance of considering HIV infection in the differential diagnosis of inflammatory polyradiculopathy, particularly when cerebrospinal fluid analysis demonstrates lymphocytic pleocytosis and imaging shows nerve root enhancement. Early diagnostic evaluation, including HIV testing, may facilitate the timely identification of the underlying infection and appropriate multidisciplinary management. Further studies involving larger patient cohorts are needed to better characterize the pathophysiology, natural history, and optimal management of HIV-associated inflammatory polyradiculopathy.
